# Methicillin-Resistant *Staphylococcus aureus* Carriage among Students at a Historically Black University: A Case Study

**DOI:** 10.1155/2013/979734

**Published:** 2013-01-20

**Authors:** Hua Shen, Eyitayo Akoda, Kunyan Zhang

**Affiliations:** ^1^Department of Biology, Virginia State University, 1 Hayden Drive, Petersburg, VA 23806, USA; ^2^Departments of Pathology & Laboratory Medicine, Microbiology, Immunology & Infectious Diseases, and Medicine, University of Calgary, Calgary, AB, Canada T2N 4N1

## Abstract

*Background*. Black people in the USA is afflicted with a higher rate of methicillin-resistant *Staphylococcus aureus* (MRSA) infection. This study determined the prevalence of MRSA carriage among black college students at a university setting. *Methods*. Hand and nasal swabs were collected and screened for MRSA by mannitol fermentation, coagulase, and DNase activities and their resistance to oxacillin. MRSA isolates were analyzed for antimicrobial resistance pattern, genetic profile for staphylococcal cassette chromosome *mec* (SCC*mec*) type, pulsed-field type, multilocus sequence type (ST), and the presence of Panton-Valentine leukocidin (PVL) gene. *Results*. MRSA was isolated from 1 of the 312 (0.3%) hand swabs and 2 of the 310 (0.65%) nasal swabs, respectively. All isolates lack multidrug resistance and have type IV SCC*mec*, characteristic of community-associated MRSA. These isolates were a ST8-MRSA-IVa-PVL(+) (USA300 strain), a ST8-MRSA-IVb-PVL(−), and a new MLST, ST2562-MRSA-IV-PVL(−), identified in this study. These isolates were thus not transmitted among students. *Conclusion*. We found a low rate of MRSA carriage among students in a black university. Our finding highlights the need of future study which involves multiinstitutions and other ethnic group to assess the association of black race with MRSA carriage.

## 1. Introduction

*Staphylococcus aureus,* a bacterium commonly isolated from humans, is an important causative agent for skin and soft tissue infections, pneumonia, septic arthritis, endocarditis, osteomyelitis, and sepsis [1]. Methicillin-resistant *Staphylococcus aureus *(MRSA) strains resistant to *β*-lactam antimicrobial agents have caused increased *S. aureus* infection and mortality [2, 3]. Black people in the USA is afflicted with a higher rate of MRSA infection as indicated by studies of pediatric patients, community-onset skin and soft tissue infections, right-sided endocarditis patients, and hospital patients [4–10]. A report by Klevens et al. [11] described significant race disparity through a surveillance program in 2004-2005: incidence rates of invasive MRSA infection were consistently higher among blacks than whites in all age groups. Overall, infection rate was more than twice as high, and mortality rate was 80% higher for blacks than for whites. The reason for these racial disparities is not known.

It is well known that *S. aureus* carriage is a major risk factor for infection, and MRSA colonization as opposed to MSSA colonization is associated with an increased risk of infection [1–3]. A question needs to be answered is whether a higher rate of MRSA carriage occurs among blacks. National MRSA prevalence studies by the National Health and Nutrition Examination Survey Program (NHANES) [12–14] found that among noninstitutionalized US population the rates for MRSA carriage were 0.9% for white and 1.1% for black in 2001-2002, and 1.6% for both whites and blacks in 2003-2004 [14]. These cross-sectional surveys identified risk factors for MRSA carriage as healthcare facility exposure, age ≥60 years' females, diabetes, and household with income below poverty level. Some of the risk factors are also disproportionally associated with the blacks, making it difficult to discern the effect of race for MRSA carriage.

 Compelled by the high prevalence of MRSA infection in black persons, we conducted a survey to determine the prevalence of MRSA carriage among students of a historically black university in 2006-2007.

## 2. Materials and Methods

### 2.1. Sample Collection

Institutional review board approval was obtained prior to the survey. This survey was conducted at a historically black university; samples were collected during a 14 months' period from February 2006 to March 2007. Three to five biology senior students enrolled in a research class each semester assisted sample collection. Participants were recruited mostly from biology general education classes to better represent the student population. Samples were collected from those who authorized letter of informed consent. With separate swabs, a single sample was collected from each of the two body sites, nose and hand, from each participant. Swabs from each participant were coded with a numeric number that was followed by a letter H for hand swab, and a letter N for nasal swab, respectively. Cultures dried in an incidence of prolonged incubation were withdrawn from this study thus not all participants' nasal and hand samples were included. Repeated samples were excluded when indicated by information of participants. Health care facility exposure and antibiotic usage 6 months prior to sample collection were inquired, but answers were incomplete and inaccurate (e.g., incorrect apprehension of antibiotic usage) thus not included in this paper. For nasal sample collection, a sterile cotton-tipped swab was moistened by inserting into sterile 0.85% NaCl saline solution and then expressed out extra liquid. The moistened swab was then inserted into both anterior nares, one at a time with the same swab, and rotated gently against the inner surface. The swab was withdrawn and immediately streaked on a mannitol salt agar (MSA, BD, Sparks, MD, USA). Skin samples from hand were collected with a moisten swab prepared as described for nasal sampling, by gently rubbing a 1 cm^2^ area of fingertips of one hand, the swab was then immediately streaked on a MSA. MSA plates were incubated at 35°C for up to 48 hours. Nasal and skin samples were collected from 364 participants, of these 347 were black students. Among the cultures lost in the incubation incidence, 35 hand and 37 nose cultures were from the black students. Data from the 312 skin and 310 nose samples from black students are analyzed in this paper.

### 2.2. MRSA Screening

One or two (if obvious difference in color) white or yellow or cream-colored colonies on MSA plate with yellow surrounding (indicative of fermentation) were subcultured on trypticase soy agar (TSA). Each subculture was subjected to Gram stain followed by microscopic examination. Only mannitol-fermenting, gram-positive cocci were screened for oxacillin resistance using oxacillin screen agar (BD, Sparks, MD) which contains 0.6 *μ*g/mL oxacillin and 40 mg/mL NaCl. Following the manufacturer's instruction, isolates were grown in trypticase soy broth (TSB) at 37°C for 4–6 hours and diluted to 0.5 McFarland standard with TSB. Then ten microliter of the inoculate suspension was deposited onto an oxacillin screen agar and smeared in a 1 inch^2^ area with a sterile cotton-tipped swab. *S. aureus* ATCC 29213 (oxacillin susceptible) and *S. aureus* ATCC 43300 (oxacillin resistant) were included for quality control. The plates were incubated at 35°C for 24 hours and observed for growth.

Isolates which formed visible colonies on oxacillin screen agar were tested for coagulase activity by tube coagulase test (Fluka Analytical, Buchs, Switzerland) using rabbit plasma with EDTA following the manufacturer's instruction. *S. aureus* ATCC 43300 and *Escherichia coli* ATCC 25922 were included as positive and negative controls, respectively. Oxacillin-resistant coagulase-positive isolates were tested for deoxyribonuclease (DNase) activity using DNase test agar (BD, Sparks, MD, USA). Isolates were transferred onto DNase test agar and incubated at 35°C for 24 hours. Following incubation, the plates were flooded with 1 N HCl. A clear zone around the area of growth indicated DNase activity. Oxacillin-resistant, coagulase-positive, and DNase-positive isolates were identified as MRSA.

### 2.3. Antimicrobial Susceptibility Pattern by Disc Diffusion Assay

Antibiotic testing was performed using standard NCCLS methods for antimicrobial disk susceptibility [15]. Antibiotics tested included penicillin (10 IU), ciprofloxacin (5 *μ*g), clindamycin (2 *μ*g), erythromycin (15 *μ*g), doxycycline (30 *μ*g), tetracycline (30 *μ*g), trimethoprim/sulfamethoxazole (1.25/23.75 mcg), and vancomycin (30 *μ*g) (BD, Sparks, MD). Briefly, MRSA isolates were grown in TSB for 4–6 hours to reach or to be diluted to 0.5 McFarland standard with TSB. A sterile cotton-tipped swab was moistened in the standardized suspension and then streaked on Mueller-Hinton agar (BD, Sparks, MD). Antibiotic discs were positioned on the surface of the inoculated plate. After incubating at 35°C for 18 hours, the diameter of zone of inhibition was measured, and susceptibility was determined according to the definition by manufacturer. *S. aureus* ATCC 25923 was included in the tests for quality control. 

### 2.4. Erythromycin Induced Clindamycin Resistance-D Test

MRSA isolates were grown in TSB at 37°C for 4–6 hours, diluted to 0.5 McFarland standard with TSB, and streaked on TSA containing 5% sheep blood. Antimicrobial discs of erythromycin (15 *μ*g) and clindamycin (2 *μ*g) were placed 17 mm apart from edge to edge on inoculated plate. The plates were incubated at 35°C for 18 hours and then photographed and visually inspected.

### 2.5. SCCmec Typing

Sequences specific for SCC*mec* type I to V and *mecA*, respectively, were detected by multiplex PCR on an iCycler (Bio-Rad) using primers (Operon, Huntsville, AL, USA) and conditions described by Zhang et al. [16]. Strains of known SCC*mec* types, JCSC4744 (type IVa) and JCSC2172 (type IVb), were included in PCR for comparison. PCR amplicons were analyzed by electrophoresis through 2% agarose gel (Fisher, Fair Lawn, NJ, USA).

### 2.6. PFGE

Isolates were genetically typed using PFGE after digestion with *SmaI* following a standardized protocol [17]. PFGE-generated DNA fingerprints were digitized and analyzed with BioNumerics Ver. 3.5 (Applied Maths, Sint-Martens-Latem, Belgium) by using a position tolerance of 1.0 and an optimization of 1.0. Cluster analysis was performed by the unweighted pair group method, using arithmetic averages (UPGMA), and DNA relatedness was calculated on the basis of the Dice coefficient. Isolates were considered to be genetically related if their macrorestriction DNA patterns differed by ≤7 bands and the Dice coefficient of correlation was ≥75% [18]. The prototypic pulsotype strains NRS 382, NRS 383, NRS 384, NRS 123, NRS 385, NRS 386, and NRS 387 for USA100, USA200, USA300, USA400, USA500, USA700, and USA800, respectively, were obtained through the Network on Antimicrobial Resistance in *S. aureus* (NARSA). 

### 2.7. MLST

Sequences of internal fragments of 7 house-keeping genes, *arcC, aroE, glpF, gmk, pta, tpi, *and* yqiL* of each MRSA isolate were amplified by PCR as described by Enright et al. [19]. PCR amplicons were purified and nucleotide sequence determined for both strands at Beckman Coulter Genomics (Beverley, MA, USA). The sequences were then trimmed so that they correspond exactly to the region that used to define the alleles. The trimmed sequences of each strain were submitted to the *S. aureus* database (http://saureus.mlst.net/) to obtain allelic profile. 

### 2.8. Detection of PVL Gene

Sequence specific for the junction region of the contiguous, cotranscribed *lukS*-PV and *lukF*-PV genes was detected by PCR with primers (Operon, Huntsville, AL, USA) and conditions described by Lina et al. [20]. PCR amplicons were analyzed by electrophoresis through 2% agarose gel.

## 3. Results

### 3.1. Survey Participants

Student population during the survey period was an average of 4,796 headcounts, of which 94% was of black race. Students' demographic source was 70% in-state and 30% out-of-state. Date of birth was collected from 211 participants between fall 2006 and spring 2007, of which 205 (97%) aged 18–25 years.

### 3.2. MRSA Carriage

Since* S. aureus* can be isolated from multiple skin locations but the nose is the primary reservoir for replication and spread to other body areas [2], we investigated MRSA carriage at nares and hand for comparison. Hands were chosen mainly because it is convenient site to sample and would less likely cause concern to students. Three hundred and twelve skin (hand) swabs and 310 nasal swabs from black students were included in this study. Of these, 1 MRSA isolate (237H) was obtained from skin swabs (0.3%), but the nasal swab of the same participant (237N) was among the dried MSA cultures in the initial screening thus not included in this study. Two MRSA isolates (81N and 240N) were obtained from nasal swabs (0.65%), but the hand swabs of these same participants (81H and 240H) were negative for MRSA. The lower rate of hand carriage than nasal carriage is in agreement with the understanding that *S. aureus* primarily colonizes the nose. Difference in MRSA carriage between male and female could not be discerned because of the small number of MRSA isolates (Table 1).

### 3.3. Antimicrobial Susceptibility Profile

All 3 MRSA isolates were found resistant to penicillin and ampicillin, two (81N and 240N) were also resistant to erythromycin, but all were susceptible to ciprofloxacin, clindamycin, doxycycline, tetracycline, trimethoprim-sulfamethoxazole, and vancomycin (Table 2). These have the drug-resistance pattern of community-associated MRSA (CA-MRSA) strains.

Clindamycin is a main choice for treating CA-MRSA skin and soft-tissue infections. Some CA-MRSA isolates, however, have the potential to become resistant to clindamycin [21]. We conducted D test for the 2 erythromycin resistant, but clindamycin-susceptible isolates strains, (81N and 240N) and found that one strain (81N) was positive for erythromycin-induced clindamycin resistance.

### 3.4. MRSA Molecular Analysis

**Figure 1:**
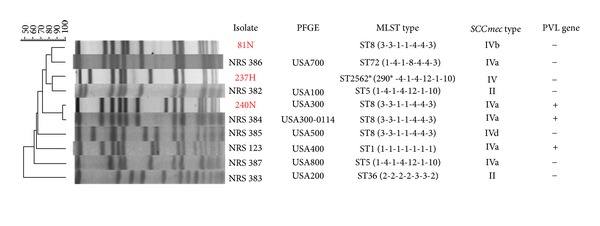
Molecular and genotypic traits of methicillin-resistant *Staphylococcus aureus* (MRSA) isolates. PFGE, pulsed field gel electrophoresis; MLST, multilocus sequence typing; SCC*mec*, staphylococcal cassette chromosome *mec*; PVL, Panton-Valentine leukocidin (+, positive; −, negative); Strains NRS 382, NRS 383, NRS 384, NRS 123, NRS 385, NRS 386, and NRS 387 are the prototypic pulsotype strains of USA100, USA200, USA300, USA400, USA500, USA700, and USA800, respectively; 290*: a new *arc*C allele with one base-pair variation from allele 1, 29G→A; ST2562*: a new multilocus sequence type identified in this study, data of MLST type, SCC*mec *type, and PVL gene of strains NRS 386, NRS 384, NRS 382, NRS 385, NRS 123, NRS 387, and NRS 383 are obtained from the website of Network on Antimicrobial Resistance *Staphylococcus aureus* (NARSA): molecular typing control strains. (http://www.narsa.net/control/member/search?repositoryId=108).

All 3 MRSA isolates carried type IV SCC*mec*, IV for 237H, IVa for 240N, and IVb for 81N (Figure 1). This is consistent with their lack of multidrug resistance.

Isolate 240N was determined to be USA300 (>95%), MLST type 8 (ST8: 3-3-1-1-4-4-3), and positive for PVL gene (Figure 1). It matches closely to the prototypic pulsotype strain NRS 384 of USA300: ST8-MRSA-IVa-PVL(+).

 Isolate 237H clusters with USA100 (82%) by PFGE. Its sequence type was determined as ?-4-1-4-12-1-10. The first locus, *arc*C, has one base-pair variation (29G→A) from allele 1, representing a novel allele. The new *arc*C allele was assigned as *arc*C290, and the new strain 237H was assigned ST2562 (290-4-1-4-12-1-10) (Figure 1) (http://saureus.mlst.net/sql/burstspadvanced.asp). 

 Isolates 81N clusters with USA700 (82%), MLST type 8 (ST8:3-3-1-1-4-4-3), and negative for PVL gene.

## 4. Discussion

SCC*mec* is a mobile genetic element that carries the methicillin-resistance gene *mec*A. Currently, eleven major types of SCC*mec* have been identified [22]. Type I, II, and III carry additional drug-resistance gene and often found in healthcare-associated MRSA (HA-MRSA) strains; type IV and V carry no additional drug-resistance gene and are CA-MRSA strains [3, 22]. All 3 isolates carry type IV SCC*mec*, characteristic of CA-MRSA.

MRSA strains have been established by PFGE as USA100 to USA1200, and multilocus sequence typing (ST) based on allelic profile of seven housekeeping genes. In this study, we identified one USA300 strain which is also ST8, a CA-MRSA strain as a primary cause for staphylococcal skin and soft tissue infection. The other 2 isolates are not so typical: 81N is a ST8 and related to USA700; 237H clusters with USA100 and was a ST2562, a new MLST strain.

 From 312 skin (hand) swabs and 310 nasal swabs, 1 MRSA (0.3%) isolate was obtained from skin swabs and 2 (0.65%) from nasal swabs. Given the high rate of MRSA infection among the blacks [4–11], the nasal carriage rate initially surprised us, a rate lower than the black and white (1.6%) as found in the national prevalence survey during 2001–2004 [14]. However, a lower rate of carriage was conceivable for the population of our study. First, our study subjects were university students aged mostly 18–25 (see Section 3.1) at the time of sample collection; therefore, some of the known risk factors such as children, age greater than 60, and educational level below high school diploma were eliminated in our study.

Further, living in crowded household and having household members of MRSA-colonized persons have been found to increase the risk of becoming colonized by MRSA [3, 23–26]. Students can be contaminated by MRSA by a household member who is infected or a carrier through person to person contact, contact of surfaces, or contaminated household items and become a carrier. Living in the university dormitory limits the students' chance of such contacts with household member. Therefore, when students lose their transiently carried MRSA strain, they may have a reduced chance to be retransmitted by a household member. 

Other studies on nasal carriage of MRSA among college students had reported varied findings. In a Hawaii college in 2005, 3 of 100 students and faculty members (3%) carried MRSA [27]; in a Texas college in 2007-2008, 15 of 203 students (7.4%) carried MRSA, and the associated risk factor was determined as hospitalization within 12 months [28]; at 2 higher-learning institutions in Tennessee in 2009, investigation of student athletes found 4 of 124 (3.2%) in one institution, and 1 of 153 (0.65%) in another, carried MRSA [29]. However, the carriage rates are difficult to compare given the range of variables and the limited sample sizes.

There have been reports describing a lower rate of nasal carriage of *S. aureus* in persons of African origin. Millian et al. in a 1960 study found that *S. aureus* carriage in African American female and male aged 18–69 were 19.5% (of 67) and 9.7% (of 72), whereas in their white counterparts were 36.7% (of 147) and 38.4% (of 271), respectively [30]. Nobel in a study of British school children aged 5–14 at an immigrant community in 1974 found nasal carriage among black children was 30% whereas among their white counterparts was 41%, a less striking but similar trend [31]. On the contrary, Lamikanra et al. in a survey of Nigerian students from primary school to university in 1985 found that *S. aureus* carriage as high as 56.4% [32]. Yet, the surveys by NHANES found *S. aureus* carriage in the USA was 25% in blacks and 33.3% in whites during 2001-2002; and 22% in blacks and 30.7% in whites during 2003-2004 [14]. This recent data indicates a more than 8% lower *S. aureus* carriage rate in blacks than in whites, lending supporting evidence to the notion of lower *S. aureus* carriage among black people.

A lower rate of *S. aureus* colonization could mean a higher risk for MRSA colonization or infection. For example, studies in the health care setting in 1960s suggested that an individual with persistent nasal carriage of *S. aureus* is generally protected against the acquisition of new *S. aureus* strains, while intermittent *S. aureus* carriers, in contrast, may be at risk of acquiring drug-resistant *S. aureus* [33]. Stevens et al. [23] found that skin infection took longer to develop in methicillin-sensitive *S. aureus* carriers (2.9 year) than MRSA carriers (0.8 years) and non-*S. aureus* carriers (2.2 years), supporting such hypothesis. Persons of noncarrier for *S. aureus* may suffer low level of *S. aureus* infection and it would be an advantage before the drug-resistance era. However, with the rise and wide spread of drug-resistant *S. aureus* the noncarrier has reduced barrier to the colonization of exposed *S. aureus* and can be at higher risk of being transmitted a MRSA strain and become infected [2]. This seems also a plausible factor for a higher rate of MRSA infection among the black population. But further investigation is needed.

Our study has several limitations. First, our results represented a convenience sample from a single institution, therefore, it cannot be generalized or used to predict prevalence in other settings. Second, we could not conduct a controlled study that would require the participants of an equivalent student population of other ethnic group. Third, it should be noted that our data was collected during 2006-2007, but MRSA prevalence data for the same period could not be found for comparison. Forth, we did not screen for methicillin-sensitive *S. aureus* which would offer additional data to understand *S. aureus* carriage among people of African origin. Fifth, there is a growing evidence that CA-MRSA may preferentially colonize other parts of the body such as throat and inguinal areas [3, 26, 34–37]. As illustrated by Miller et al. [26] in a survey of 3 body sites, nasal, oropharynx, and inguinal areas, nasal sample only revealed 49% of MRSA carriage of the combination of the 3 body sites. In the absence of additional oropharynx and inguinal specimens, the rate of MRSA carriage in our study was certainly underestimated. Our finding nonetheless highlights the need of future study which involves multiinstitutions and other ethnic group to assess the association of black race with MRSA carriage.

In conclusion, our study found nasal carriage rate of MRSA among the black college students was 0.65%, and skin carriage in hand was 0.3%, a lower nasal carriage rate than that found among whites and blacks by the NHANES surveys. All three isolates are CA-MRSA as indicated by type IV SCC*mec* and nonmultidrug resistance. Their distinct molecular profiles do not indicate transmission among students.

## Figures and Tables

**Table 1:** Methicillin-resistant *Staphylococcus aureus* carriage among black university students, 2006-2007.

Participants	Skin (hand)		Nasal	
	Tested No (%)	MRSA No (%)	Tested No (%)	MRSA No (%)
Female	176 (56.4)	1 (0.6)	176 (56.8)	1 (0.6)
Male	136 (43.6)	0	134 (43.2)	1 (0.7)

Total	312 (100)	1 (0.3)	310 (100)	2 (0.65)

**Table 2:** Antimicrobial susceptibility of methicillin-resistant *Staphylococcus aureus* isolates.

MRSA isolates	Antibiotics tested								D test
	PE	CI	CL	DO	TE	TS	VA	ER	
81N	R	S	S	S	S	S	S	R	+
237H	R	S	S	S	S	S	S	S	NA
240N	R	S	S	S	S	S	S	R	−

PE: penicillin (10 IU); CI: ciprofloxacin (5 *μ*g); CL: clindamycin (2 *μ*g); DO: doxycycline (30 *μ*g); TE: tetracycline (30 *μ*g); TS: trimethoprim/sulfamethoxazole (1.25/23.75 mcg); VA: vancomycin (30 *μ*g); ER: erythromycin (15 *μ*g); Quality control strain was *Staphylococcus aureus* ATCC 25923; D Test: test of erythromycin induced clindamycin resistance; NA: non-applicable.
